# Patient e-Visit Use and Outcomes for Common Symptoms in an Integrated Health Care Delivery System

**DOI:** 10.1001/jamanetworkopen.2021.2174

**Published:** 2021-03-24

**Authors:** Reena Bhargava, Gregg Gayre, Jie Huang, Evangeline Sievers, Mary Reed

**Affiliations:** 1 The Permanente Medical Group, Santa Clara, California; 2 The Permanente Medical Group, San Rafael, California; 3 Division of Research, Kaiser Permanente, Oakland, California; 4 The Permanente Medical Group, Oakland, California

## Abstract

This cohort study examines whether e-visits can adequately treat patients who have medical concerns or whether follow-up care is typically still needed within 72 hours or 7 days.

## Introduction

Electronic physician visits, or e-visits, use a digital virtual care platform with structured, asynchronous exchange between patients and clinicians to provide care without scheduled appointments.^1^ e-Visits can be an efficient model for receiving care during pandemics such as coronavirus disease 2019 (COVID-19).^2^

Through a patient portal, e-visits collect clinician-developed, internet-based questionnaires for physician management with clinical decision support tools and electronic health record (EHR) order sets. Little evidence exists on characteristics of patients using e-visits and success in managing patients’ concerns. We evaluated patients’ adoption and success of primary care e-visits by monitoring the 7-day follow-up care needed within an integrated health care delivery system.

## Methods

This cohort study was approved by the Kaiser Permanente Northern California institutional review board, which waived the requirement for written informed consent because it is a data-only study. This study follows the Strengthening the Reporting of Observational Studies in Epidemiology (STROBE) reporting guideline for cohort studies.

In Kaiser Permanente Northern California, e-visits for 5 common adult primary care concerns—upper respiratory infection (URI), emergency contraception (EC), conjunctivitis, pharyngitis, and urinary tract infection (UTI)—were added to the patient portal by 2019. Patients completed concise e-visit questionnaires about their symptoms (up to 30 questions, depending on number of symptoms and concerns) with exclusion and inclusion criteria. The conjunctivitis e-visit allowed uploading of digital pictures and choosing the photograph best matching their symptoms.

A primary care physician reviewed patients’ e-visit submissions and responded electronically within 2 hours using clinical decision support tools and electronic order sets for prescriptions, laboratory tests, diagnoses, and EHR smartphrases, with a personalized message including advice sent to the patient via a secure message portal. e-Visit questionnaire responses and physician’s plan of care are automatically included in the patient’s EHR.

We examined adoption of newly developed e-visits between July 1 and December 31, 2019, including demographic characteristics of all patient users. We assessed e-visit success outcomes by evaluating follow-up care seeking within 72 hours and 7 days after the e-visit, including associated outpatient appointments (office, video, telephone) or emergency department (ED) visits and any secure email messages.

We used the χ^2^ test to test for differences in patient characteristics across e-visit concern type. Testing was 2-tailed, and the level of statistical significance was set at *P* < .05. Data analysis was performed with SAS version 9.4 (SAS Institute) from January to November 2020.

## Results

Among 21 070 total patient e-visits (Table), 17 014 (80.75%) were women, 10 728 (50.92%) were White, and they had a mean (SD) age of 36 (12.6) years. Of all e-visits, 12 986 (62%) had prescriptions ordered, with cough suppressants for 72% of URI e-visits (5660 of 7910) and antibiotics for 19% of pharyngitis e-visits (881 of 4691) and 81% of UTI e-visits (5118 of 6297) (Figure). Patient characteristics varied significantly by e-visit concern (all *P* < .001, Table). Within 7 days, 13.5% (2841) of e-visits had an associated outpatient appointment, 0.8% (176) had an ED visit, and 4.5% (957) exchanged a secured message (ranging from 1.6% [17 of 1069] for EC to 5.7% [268 of 4691] for pharyngitis).

**Table.** Patient Characteristics and Follow-up Events

Patient characteristics^a^	Patients, No. (%)^b^					
	All (N = 21 070)	URI (n = 7910)	UTI (n = 6297)	Pharyngitis (n = 4691)	Conjunctivitis (n = 1103)	EC (n = 1069)
Age, y						
18-24	3016 (14.3)	688 (8.7)	1223 (19.4)	652 (13.9)	79 (7.2)	374 (35.0)
25-29	3965 (18.8)	1276 (16.1)	1246 (19.8)	908 (19.4)	164 (14.9)	371 (34.7)
30-34	3945 (18.7)	1522 (19.2)	1074 (17.1)	953 (20.3)	212 (19.2)	184 (17.2)
35-39	3094 (14.7)	1269 (16.0)	752 (11.9)	783 (16.7)	199 (18.0)	91 (8.5)
40-49	3666 (17.4)	1639 (20.7)	959 (15.2)	804 (17.1)	219 (19.9)	45 (4.2)
50-64	2557 (12.1)	1160 (14.7)	768 (12.2)	466 (9.9)	159 (14.4)	4 (0.4)
65 and older	827 (3.9)	356 (4.5)	275 (4.4)	125 (2.7)	71 (6.4)	0
Women	17 014 (80.7)	5610 (70.9)	6295 (100.0)	3263 (69.6)	777 (70.4)	1069 (100.0)
Men	4056 (19.3)	2300 (29.1)	2 (0.0)	1428 (30.4)	326 (29.6)	0
Race/ethnicity						
White	10 728 (50.9)	4205 (53.2)	3230 (51.3)	2421 (51.6)	618 (56.0)	254 (23.8)
Black/African American	1461 (6.9)	450 (5.7)	451 (7.2)	319 (6.8)	65 (5.9)	176 (16.5)
Hispanic	4823 (22.9)	1636 (20.7)	1461 (23.2)	1094 (23.3)	220 (19.9)	412 (38.5)
Asian	362917.2	1427 (18.0)	1051 (16.7)	767 (16.4)	183 (16.6)	201 (18.8)
e-Visit prescribing^c^						
Prescription ordered	12 986 (61.6)	5660 (71.6)	5118 (81.3)	881 (18.8)	492 (44.6)	835 (78.1)
After e-visit, 72 h follow-up^d^						
Secure message	734 (3.5)	302 (3.8)	160 (2.5)	220 (4.7)	40 (3.6)	12 (1.1)
Any related outpatient visit	452 (2.1)	0	452 (7.2)	0	0	0
Physician office visit	158 (0.7)	0	158 (2.5)	0	0	0
Telephone visit	289 (1.4)	0	289 (4.6)	0	0	0
Video visit	5 (0.0)	0	5 (0.1)	0	0	0
ED visit	111 (0.5)	36 (0.5)	42 (0.7)	25 (0.5)	3 (0.3)	5 (0.5)
After e-visit, 7 d follow-up^d^						
Secure message	957 (4.5)	381 (4.8)	241 (3.8)	268 (5.7)	50 (4.5)	17 (1.6)
Any related outpatient visit	2841 (13.5)	903 (11.4)	612 (9.7)	1137 (24.2)	105 (9.5)	84 (7.9)
Physician office visit	1440 (6.8)	337 (4.3)	267 (4.2)	758 (16.2)	43 (3.9)	35 (3.3)
Telephone visit	1,338 (6.4)	538 (6.8)	339 (5.4)	355 (7.6)	57 (5.2)	49 (4.6)
Video visit	63 (0.3)	28 (0.4)	6 (0.1)	24 (0.5)	5 (0.5)	0
ED visit	176 (0.8)	73 (0.9)	46 (0.7)	45 (1.0)	5 (0.5)	7 (0.7)

Abbreviations: EC, emergency contraception; ED, emergency department; URI, upper respiratory infection; UTI, urinary tract infection.

^a^ Patient characteristics show demographic characteristics (extracted from the patient’s electronic health record) of patients initiating an e-visit through the patient portal. We used the χ^2^ test to test for differences in patient characteristics across e-visit concern type at a 2-tailed .05 level of statistical significance.

^b^ *P* < .001 for all differences in patient characteristics by specific e-visit concern type.

^c^ e-Visit prescribing shows e-visit concern–related prescription orders linked with the e-visit. Cough suppressants were prescribed for URI (cold, cough, or flu) e-visits, and antibiotics for pharyngitis (sore throat) and UTI.

^d^ Post–e-visit follow-ups show further interactions with the health system within 72 hours and within 7 days after an e-visit. ED visits and secure message measures include for any clinical reason within 72 hours or within 7 days after an e-visit. Secure messages for pharyngitis concerns may be generated by strep test follow-up.

**Figure.**
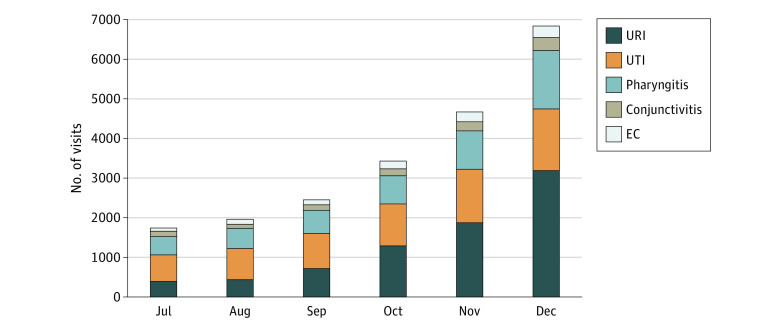
Number of e-Visits from July to December 2019 EC indicates emergency contraception; URI, upper respiratory infection; and UTI, urinary tract infection.

e-Visits took 2 to 3 minutes of physician time. No quality concerns were identified during random medical record reviews of follow-up ED visits.

## Discussion

We studied the early adoption of e-visits in an integrated delivery system and found e-visit users were predominantly less than 40 years old and female.^1,3^ e-Visits may have particular usefulness in less-engaged patient groups. We can conclude that most patients were successfully managed with asynchronous digital care since 81% did not seek follow-up care within 7 days, a rough proxy for treatment success.^4^

Distinct from computerized chatbots and symptom checkers available in direct-to-consumer applications that are not financially reimbursable,^2^ physician-developed e-visits provide efficient direct physician care integrated with patient EHRs.^4^ To our knowledge, this is the first study describing the adoption and success of e-visits in clinical care delivery for several symptoms. Still, limited reimbursement for digital services is a barrier to clinician adoption.^2^ e-Visits took 2 to 3 minutes of physician time, offering greater flexibility than scheduled telehealth or office visits and efficient care delivery.

This study had some limitations. One limitation was the lack of statistical comparison with traditional physician visits. Another limitation was the inability to assess patient symptoms independent of care seeking.

e-Visits offer quick, safe patient access to virtual health care for specific conditions without needing a scheduled visit, transportation, or time off work. This study’s results suggest that a predominance of e-visits delivered clinical care successfully without follow-up visits or messages. Further research about continued adoption, especially during shifts to virtual during the COVID-19 pandemic, is needed.
